# On the Real Merits of Theophrastus Von Hohenheim [Paracelsus.]

**Published:** 1842-07

**Authors:** 


### ART. XIII.

*Zur Würdigung des Theophrastus von Hohenheim.* Von Dr. KARL FRIED. HEIN. MARX, Hofrath und Professor an der Georg-August-Universität.—*Göttingen*, 1842. 4to, pp. 140.

*On the Real Merits of Theophrastus von Hohenheim,* [*Paracelsus.*] By Dr. MARX, Counsellor and Professor in the George-Augustus University of Göttingen.—*Göttingen*, 1842.

PROFESSOR MARX has been long recognized in Germany as one of the most erudite men in that country of scholars. And yet, although his works are numerous, he is scarcely known in England except as the author of a treatise on Poisons, and another on Contagion.* We believe, however, that our unacquaintance with his singular merits as a profound scholar, a skilful physician, and a most amiable man, has reached its term, and that henceforth no name in German medical literature will be better known by the medical profession in this country than that of Dr. Marx.

The work now before us is one which will raise the reputation of Dr. Marx, as an industrious and enlightened scholar and medical historian, to the very highest rank. It is indeed a stupendous monument of industry and learning, and cannot fail to be appreciated as such by the learned men of every country. This work was first read, in the form of memoirs, before the Royal Society of Göttingen, and is now reprinted from its Transactions. Slender though the volume be, its composition occupied its author a long time; and well it might, when the incredible mass of books necessary to be consulted is considered. Full half the bulk of the volume consists of mere references to these works; and the comparison of the text with the notes sufficiently proves that they were not merely referred to, but actually read and digested. The consequence has been that we have now, for the first time, a thorough sifting of the life and writings of Paracelsus, and an appreciation of his merits, which may not merely be received as correct, but must be almost regarded as *final*, if we may so speak, inasmuch as the results obtained are of a kind that can scarcely be invalidated, much less set aside, by any subsequent researches.

In the present article we shall present our readers with an abridged outline of the more remarkable facts and doctrines detailed in this extraordinary production; and in doing so, as well with the view of economizing space as of ensuring accuracy, we shall avail ourselves often of the author's own words, or of those of the singular subject of his memoir,

* The following works of Dr. Marx are now before us:

1. Diatribe de Structura atque vita venarum.—Carlsruhæ, 1819. 8vo, pp. 104.
2. Göttingen in Med. Phys. u. Histor. Hinsicht geschildert.—Göttingen, 1824. 8vo, pp. 392.
3. Origines Contagii.—Göttingen, 1824. 8vo, pp. 202.
4. De Euthanasia Medica.–Göttingen, 1826. 4to, pp. 16.
5. Die Lehre von den Giften. Band i.—Göttingen, 1827. 8vo, pp. 270.
6. Die Lehre von den Giften. Band ii.—Göttingen, 1829. 8vo, pp. 580.
7. Allgemeine Krankheitslehre.—Göttingen, 1833. 8vo, pp. 273.
8. Grundriss zur Lehre von der Krankheit und Heilung.—Carlsruhe, 1838. 8vo, pp. 447.
9. Zur Lehre von der Lähmung der untern gliedmassen.—Carlsruhe, 1838. 12mo.
10. Herophilus: Ein Beitrag zur Geschichte der Medicin.—Carlsruhe, 1838. 8vo.
11. Zum Andenken an J. F. Blumenbach.—Göttingen, 1840. 4to.

scattered through the text or given in the notes. We shall seldom interrupt the narrative by any comments of our own, but leave the impression on the reader's mind to take the form which the perusal seems calculated to give.

Theophrastus von Hohenheim, better known under the name of Paracelsus, shared the lot of many men of genius and great reformers, only to be appreciated long after his death. The cause of this is too obvious to be dwelt on.

"Opinions," says Dr. Marx, "which have been prevalent during centuries, or which have been promulgated with much confidence by the leaders of the opinion of the day, are successively stamped as infallible; and it requires a conviction based on no ordinary grounds to raise doubts against established views, or such as are supported continually by specious argument. It is difficult to believe that which has been hitherto considered as true to be but illusion and fallacy. I myself had indulged in the usual erroneous opinions relating to Theophrastus, and it was not until I had read his writings, studied the history of his times, and had duly weighed the opinions of his contemporaries and others, that I arrived at conclusions quite different from those which have hitherto prevailed. On this subject there is not to be found in history another man who has been so much misunderstood as Theophrastus. His very name (*Bombastus*) has become the by-name of any extraordinary assertion. The most strange and contradictory opinions and doings were ascribed to him; and far from being judged according to the standard of his age, his real deserts are either not acknowledged or wrongly interpreted, and results which he neither called forth nor intended are laid at his door. It is the scope of this work to rectify these opinions, and to represent the man in the full light of his age and in his individual life and activity.

"The name of a man," continues Dr. Marx, "is in itself generally a matter of indifference; but it is otherwise in the present case. Many believe that nothing is needed but the mere name of the man to indicate what he really was—Philippus Aureolus Theophrastus Paracelsus Bombastus ab Hohenheim; and they argue that his whole quack-like vanity is displayed in it. But it is to be observed that he himself never used these names, neither was he so called by any of the authors who were more or less connected with him, and it was only the envy, hatred, malice, and uncharitableness of his enemies that were gratified in this strange compound of appellations." (pp. 1-2.)

In the subsequent pages Professor Marx traces the origin of these names. Some were appellatives of the noble family to which Theophrastus belonged; others originated in the peculiarities of his time, it being then the custom for men of eminence to indulge in a variety of pseudonymes, of which Calvin, for instance, had seven. As the term *bombastic*, unquestionably derived from Paracelsus, has become universally the epithet of any extravagant style of writing or speaking, it may be interesting to know that Bombast was one of the surnames of the Hohenheim family, there having lived in Würtemberg persons of that name up to the middle of the sixteenth century. (p. 5.) Our author also traces to their origin and explains the other names of Theophrastus; but with these details we need not detain our readers.

Dr. Marx begins the examination of the merits of Theophrastus with the following passage:

"It happened to Theophrastus during his lifetime, and still more so after his death, that he was proclaimed either as the first physician and philosopher, the leader of modern medicine, or as a superlative quack, pietist, imbecile, and medical heretic.* We judge men in general by their actions, and authors according to their writings; but in order to be able to do this with any degree of propriety, we have to be first convinced that the former have been related faithfully and that the latter are genuine. If, therefore, a careful sifting of things be always necessary, how much more is it so if doings the most extraordinary are attributed to the same individual, and opinions the most contradictory assigned to the same author! In order to prove at once the fertility and oddity of his mind, the falsity of his reasoning, and the confusion of his ideas, it seemed sufficient to point at the astonishing variety of his writings and the vast number of volumes collected and published under his name in folio and in quarto. But those works, which were considered as supplying authentic data for the formation of a just opinion of his merits, have caused the greatest misapprehensions and mistakes; and it would be difficult to find such another example in history, where the good name of a man has been buried under the weight of books and opinions falsely attributed to him. In the first place it is to be considered that the old editions of his works which we possess were not published by Theophrastus, and that it is even doubtful whether any work at all of his was published during his life; or if this was the case, the instances must have been rare. Another important question will be, how it happens that in those works which, on both external and internal evidence, we must attribute to him, at the side of the most pregnant and profound ideas we so often find empty and unintelligible dreams and fancies, and along with the most clear and impressive language we read the most unmeaning and incomprehensible stuff? It is only by penetrating into and appreciating the essential circumstances of his life, the mainsprings of his actions, and the spirit of his age, furthering, assisting, or impeding him, that we shall be enabled to resolve these questions in a satisfactory manner." (p. 10.)

Professor Marx begins his interesting Defence by stating that the external circumstances of Paracelsus were mostly of such a harassing nature that he could not have found leisure to give to his writings the necessary polish; and if, moreover, the MSS. (as seems often to have been the case) were badly written, they were afterwards printed with numberless and most damning typographical errors. At first the oddity and extravagant character of these writings made them to be greatly sought after; subsequently and consequently they were manufactured, as it were, on speculation. Such MSS. were, moreover, for the most part, in the possession of amateurs, as the savants and medical men of those times did not value them, or even thought themselves excused for keeping them in the background, *as not existing*. In this Theophrastus shared but the fate of most reformers; his bold and penetrating assertions, his vindication of the vernacular language as proper for the composition of medical and scientific works, made him a stumbling-block for the many who only exist by the remastication of old materials and a sturdy adherence to the traditional and received. Centuries were required to elevate the scientific alluvium to the height of such a Colossus. "However," continues our author, "the more that traditionary principles of medicine and general dogmatism made way for newer and more pregnant views, the higher rose from out the low level of this transition-epoch of the old to the new times the name of the man who, a contem-

* *Dessenius* in his work, Medicinæ veteris et rationalis defensio. Colon. Agr. 1573, 4to, p. 202, says of him: "Magus monstrosus, superstitiosus, impius et in Deum blasphemus, mendacissimus, nefandus impostor, ebriosus erro, monstrum horrendum." Morhof, in his Polyhistor. (lib. i. cap. 15, § 16,) says on the other side: "Mirabile huic homini, uti nomen, ita ingenium fuit, novus quasi literati Orbis Cometa."

porary of great reformers, had himself contributed a large share to the regeneration of knowledge." (p. 11.)

The attempts of foreigners to translate the works of Paracelsus have never had any conspicuous result; they could not grasp the wild rambling of his ideas; the more respectable amongst them soon gave up the attempt, and consequently his works and his fame soon fell altogether into the hands "of madmen or idiots." His countrymen began to collect his works soon after his death. The first collection, published from 1568-1573, by *Gerard Donn*, is very rare. *Huser* published one at Basle in 1589, in 10 vols. 4to. The same editor published another edition in 1603-1605, in 3 vols. fol. Incorrect and mutilated translations into Latin, for instance by Dr. Zacharias Parthenius, Francof., 11 (?) vols. 4to, also appeared. It is quite evident that Theophrastus did not, and could not, write *so much*, because, although it appears that he wrote and dictated a great deal, he says in the introduction to his *Grosse Wundarzneykunst* (Great Surgery), in his usual eccentric style: "If truth lies in length, Christ has said too little. It is truth which we shall write and expound, and if there be doubt or a want of reasons, we shall forbear from writing, taking an example from the prophets and evangelists, who wrote brief, the cause being that they wrote truth."* Paracelsus seems to have been very fond of dictating; and this he did so rapidly that, conjointly with other extraordinary acquirements, it led to the then belief that he was possessed by demons. We read in the *Vita Oporini*, who was his amanuensis: "Ad dictata excipienda excitabat: quæ tam expedite recitabantur, ut dæmonum instinctu ea suggeri Oporinus se putasse sæpe affirmaret."

In the detailed enumeration of the impediments thrown in the way of Paracelsus, our author mentions (p. 14) the Imperial College of Censorship, which waged war against him, as well as against all the Reformers of those times, until he was protected by the Estates (parliament) of Carinthia, to whom he dedicated some of his works. It would be incompatible with the scope of this notice to relate all the impediments which were thrown in his way; but a summary knowledge of them may be conveyed in the popular expression that the whole age *ran a muck* at him. Universities, learned societies, governments, corporations, physicians and surgeons, clergy and laymen, and last not least, even *Savants* by profession—all condemned his opinions, whilst the latter did not scruple to confess that they had not studied his works, and were not inclined to do so! So say the great Conrad Gesner (Epist. ad Crato a Crafftheim, p. 1:) "Theoph. Paracelsi errorum meministi: et petis ut mittam ad te Catalogum ejus scriptorum: quem ego certe non habeo: neque curavi ut haberem, et si facile potuissem, cum illum plane indignum cujus inter bonos scriptores mentio fieret, judicarem. Bonos dico, non solum eruditos, sed Christianos et pios saltem civiliter, sicut et Ethnici fuerunt. Theophrastus vero certe impius homo et magus fuit, et cum dæmonibus communicavit." (p. 18.)

In the following pages Dr. Marx sifts the genuine writings of Theophrastus from the spurious, and comes to the conclusion that the following are alone genuine; and he makes the general remark, that only the writ-

* Such and similar passages may be the reason that Theophrastus incurred also the hatred of clerical men, who charged him with heretic opinions.

ings on medicine or natural history are those which may be fairly ascribed to him. The following list will be interesting to the curious in medical history and bibliography:—

I. Seven books, De gradibus et compositionibus receptorum.

II. Little Surgery (*Kleine Chirurgie*), or books on French disease, &c.

III. Seven books on open ailments (*Offenen Schäden.*)

IV. Three books on the French disease (*Frantzosen*): of the impostures in treating it, of the correction of the medicines hitherto used, and of the recovery and treatment of diseases occasioned by it. This work was printed separately at Nürnberg, in 1530, in 4to.

V. Of the Impostures of Physicians. This work he intended to publish in 1520 at Nürnberg, but the Censure prevented it.

VI. Opus Paramirum; published 1562, at Mühlhausen. (Much of its oddity appertaining probably to the editor.)

VII. On the Baths of Pfeffer, (in Switzerland,) printed 1571, at Strasburg, in 8vo.

VIII. Great Surgery. (*Grosse Wundarzney.*) This is Theophrastus's *opus magnum*. He dedicated it to King Ferdinand the First of Germany. The original edition (Augsburg, 1536, fol.) seems to be very rare.

IX. Nine books, De natura rerum.

X. Three books, Exculpation of medical slander; errors and labyrinth of physicians; on the origin of gravel and stone. Written in 1538.

The following works are also, with some probability, ascribed to him: De morbis e tantaro oriundis. Scholia et observationes quædam. Tract on the Plague. Perhaps there is no better proof how far the renown of Theophrastus extended, than the fact of there being in the library of Gotha a translation of several of his works into Arabic.

In the succeeding pages Dr. Marx endeavours to exculpate the oddity of the titles of Paracelsus's works, and the strange, strong—nay, what may be called, vulgar turn of his style. Still, every one acknowledges that he never wrote a word which could be construed as offensive to morals, or even to the conventional customs of society. Amongst the things most objected to him was the great use he made of new-fangled words, but he himself meets this objection by saying, "they quarrel with me, in that I write differently from what their own writings are; the cause, however, lies not in my ignorance, but in theirs. Because I stand alone, and am new, and write German, is no reason why you should scorn my writings! I use strange words on account of the strange nature of my science. And who shall gainsay, that if there start up something new, it should not bear also a new name?" (Kl. Chir. p. 250.)

Amongst the most false and barefaced accusations against Paracelsus is to be ranked the imputation of alchemistic and theosophic fancies, and the holding him up as the representative of prejudice, astrology, spagiric medicine, magic, sorcery, and mysticism, the very things against which he most forcibly set his face.

"The period in which he lived," says Dr. Marx, "part of the sixteenth century—was one in which the light of knowledge emerged but gradually from great obscurity, when the belief in witches and demons was commanded, as it were, by the ordinances of the church, and confirmed by the decisions of courts of justice. If, then, any man, rivetted to such narrow and confined ideas, had really brought this tribute to the frailties of his times, it certainly would not afford any particular ground of inculpation; but Theophrastus, *on the contrary*, opposed these views with all his might, and endeavoured to trace out the way for ideas more enlightened." (p. 33.)

We are sorry that space does not allow us to enter any further into our author's details, most valuable as they must be to all who take an interest in medical history, and in the progress of the human mind.

In the second part of the memoir, Professor Marx investigates the medical career and the medical doctrines of Theophrastus. How and where he lived upto his thirtieth year, is scarcely known, but it appears that he passed his earlier days in continual travels and rambles. Wherefore he says, "Art does not go after any one; it is we who must follow her. I have somewhere heard that a physician ought to be a rambler, (*Landfahrer*,) and it is just that which I like best. Diseases travel everywhere over the wide world, and the doctor who would know much must travel much. Can skill be learned at the stove-side as well as by wayfaring? I guess not so. Whoever will indagate nature must trample her book with his feet. Book-learning is got by letter and letter, but nature is known by land and land—for each new land a new page. This is the line *codex naturæ*—thus must we turn her leaves." (p. 58.) Settled, however, we find Theophrastus at length at Basle, whence he issued a programme, dated 5th June, 1527, announcing his intention to lecture on medicine, physic, and surgery. He became also the city physician, and remained there two years. Although his lectures were at first highly popular, and attended even by men of established fame, his opponents (he calls them men, whose knowledge consists in wearing white gloves) succeeded in making him gradually unpopular. "It was especially his simple, rational, and conscientious way of prescribing, which became a source of much annoyance to him." (p. 54.) It was, therefore, that very simplicity of medical prescription, which is a happy characteristic of our present medical science, which made him the stumbling-block of his contemporaries, whose fame and income he seemed to encroach upon by the efficacy of his new method. Not untruly, and certainly with a pith and frankness which belong not to our skipping times, he says, "the longer the scribbling the shorter the intellect; the longer the recipe the less of virtue is in it. . . . Don't be astonished that I write such short prescriptions, the reason thereof being, that whatever might be added would d—n the medicine." Such assertions could not but raise against the poor man the whole wrath of every empty-headed quack, whose ill-gotten gains were thus at stake. His cures are stated to have been astonishing. Among them is related the case of a certain patient who had been given up by the faculty. Theophrastus prescribed for him one day, and invited him to dinner the next.

Of his travels much is said, but whether true or false cannot be known. It is stated that he had passed ten years of his life in Arabia and the adjacent countries; and when we consider what extensive (miraculous, as it was then considered,) use he made of opiates, it may be imagined that he became conversant with the heroic qualities of this drug amongst the Orientals. His travels even in Europe must have extended beyond the beaten track, as it is certain that he had visited many French and Italian universities, and even Stockholm and England. He died at Salzburg on the 24th of September, 1541, at the early age of 48.

Theophrastus paid much attention to chemistry; and, it is stated, that his premature death was occasioned by the continual inhalation of the noxious vapours of his laboratory, in which he was seen continually occupied in multifarious experiments and operations. It was a matter of controversy with his contemporaries, whether he derived his great wisdom and art from God or the Devil. Others said, that he derived much knowledge from the initiation into secret societies. It is known that such really existed at that period, but its members were tied down by an oath not to reveal their secrets. Agrippa says in his work, De Vanitate Scientiarum, "Permulta adhuc de hac arte dicere possem, nisi juratum esset de silentio." (cap. xc.)

As Paracelsus declared in many parts of his works that he had founded a new school of medicine, which he called his monarchy (*meine monarchey*), his opinions on medical men and philosophers are interesting. These are collected and recorded with astonishing industry by Dr. Marx, but we can only find room for one or two specimens. He says of Aristotle, that the very foundation of his doctrine is wrong; that Dioscorides wrote on plants, but never tried their virtues; to call Galen a prince of medicine, is wrong; the Arabs are monsters, Avicenna being a mere compiler; Albertus Magnus philosophized without experience; Arnold of Villanova gets sometimes on the right path, but soon leaves it. Amongst the recent physicians, he praises Marsilius Ficinus as the best amongst the Italians; Johannes de Vigo did not blush to lie. Of his own powers and exertions he entertained the highest opinion. "They say, that my Physica, my Theoretica, my Practica, are strange, new, wonderful, unheard-of: how can I appear otherwise than strange to those who have never walked in the sunshine?"

In the succeeding pages Dr. Marx dwells upon and explains yet farther some of the strange imputations cast from all sides and quarters on Theophrastus; some charged him with sensuality, others denied him virility; some said he derived his knowledge from demons, others called him an idiot. The particulars of these charges the lovers of medical history may refer to in the work itself: we can only find room for Dr. Marx's closing remarks, which give a very different estimate of his merits and character:

"In appreciating the character of Theophrastus we must ever recollect the period in which he lived, the persons he came in contact with, his unsteady life, his extensive travels, his early death. Assuredly with his departure his monarchy, as he called it (*seine monarchey*), fell to pieces. No one arose through him or after him to emulate his power and spirit, or to raise higher the edifice which he had founded and sketched, and for which he had collected the materials; for it would be idle to speak, in this respect, of those his hangers-on and repeaters, who were only capable of babbling empty words and formulæ, or catching the mere chaff of his writings. Consequently, he appeared as a meteor, glancing for a moment on the horizon of his age, and then vanishing without a trace. And it was left for later generations to recognize in him no idly-burning *ignis fatuus*, but a star fraught with living light and heat and the pregnant germ of a legitimate and progressive development." (p. 86.)

The third part of Dr. Marx's work is devoted to the investigation of the especial merits of Theophrastus as a medical man. The following is a brief summary of Theophrastus's exertions:

"His characteristics were,—a depth of thought, a capacity of appreciating the essential, a power of discriminating the real relations of human affairs. How much soever he had endeavoured to study nature by way of observation and experiment, it cannot escape us, that with him the individual has but a subordinate value, and that his object is rather to attain to, and to establish ideas, principles, general views, and, as it were, to transmute the sensual into the spiritual. He wishes that the physician should divine the mysteries of disease by reflection and comparison; that he should place every well-established fact in its appropriate position in the great circle of phenomena, and assign to it (the fact) by adequate and impartial judgment, a fixed relative value in the vast stock of knowledge. Not seldom does he address himself, with pithy adage, to our sympathizing, humane feelings, and calls upon the medical man most forcibly to consider his vocation to be that of a careful and soothing helper (*Helfer*). He does not afford instruction by which we may attain, step by step, easily and regularly, to the knowledge of the needful and useful, and become familiar with the systematic commonplaces of the profession; we often miss in him the regular teacher; but on the other hand, we find ourselves carried to heights, where astonishment seizes us at the views obtained; we become transported into regions where luminous flashes of thought and surprising analogies make us forget the want of merely positive information." (p. 88.)

For the sake of showing the merits of Theophrastus the more clearly, our author devotes a few pages to the state of German medicine at that period. The physicians of that country were divided into sects, Scotists, Thomists, Albertists, &c. and "cavilled at words." Up to the year 1520, scarcely any classical medical books were printed. If there had been any demand for them they would have been printed, because they were well known, but it was only books containing instructions on the preservation of health, and collections of recipes, which paid, and they could not be printed fast enough. The instruction in the universities was scanty. Anatomy was taught after Mundinus, and pigs were dissected for demonstration. The best school of surgery was at Strasburg; and amongst the men there educated, Hieronymus Braunschweig enjoyed the greatest fame as an author, as well as for his course of chemical operations. Johann von Gersdorf was also a distinguished teacher at the same school. Still, when we compare all the writings of these men, and indeed all the other works then extant, with the contents of Theophrastus's genuine writings, "we will be astonished by the comprehensiveness of his views, as well as by many new facts related, and also by the pertinent and simple observations made thereon." (p. 90.) Theophrastus had a high idea of the vast extent of medical science, but he maintains that its different departments were united by an essential connexion, and could not be fathomed singly. The philanthropic ideas of Theophrastus were in like manner far beyond the cold spirit of his age. "The highest thing," he says, "which a physician possesses is art, then love and hope. Love teacheth the art, and hope giveth the right confidence. The medical man should be mild, true, serious, and reserved in speech. But genuine art does not consist in knowing, but in doing and accomplishing." (*Grosse Wundarz.* l. 1, c. 1.)

Anatomico-physiological knowledge did not exist then in Germany, and there is scarcely a trace of it in Theophrastus's writings. But these contain a large store of medical axioms, many of which are really astonishing for the time he lived in. We will here transcribe a few of his opinions at random. He says, that for the elucidation of internal disease, anatomy is not essential, as the knowledge of the structure of the brain would never explain epilepsy; still Theophrastus acknowledged its importance in cases of surgery. He compared the general integuments to a bladder, which perspired some part of its contents by the pores; and states that, by its secretions, the physician may judge of the disease. Menstruation is an excretion of the uterus. Blood attracts nourishment, digests it within itself, and expels the heterogeneous parts. The vessels in the lungs are, as it were, the stomachs of the organ ; in them the pure is divided from the impure; what agrees with them they keep, the remainder they throw out by their tubes through the mouth. Not all heat comes from the heart, but every limb has its heat from itself. The child is developed in the womb of the mother like a germ of a plant in the soil, which passes from one form into another. The secret force which matures every organism to perfection, Theophrastus calls *archäus*, or *adech*. "It is the inside smith who hammers all right out of his own iron; the labourer who, without man's participation, works his own devices." (p. 98.)

General pathology, or as it was formerly called, the philosophy of disease, was one of the favorite topics of Theophrastus. The following are some specimens of notions in this department:

"In every disease, there is to be observed the aberration of nature, or the manner in which nature transgresses the fixed limits; then the hidden essence of the disease, and, lastly, the time, of which no one heretofore had thought. Disease is a very relative state: As there are various degrees of softness in silver, and yet every kind is silver, so is it with the alteration of health. It is as hard to find perfect health as a piece of earth without a weed; disease is essentially immaterial, and as little to be laid hold of as the wind; we must not then expect to remove it by mere material means. Every disease does not manifest itself openly, but frequently conceals itself under a foreign aspect; and this the doctor should well look to. Disease commonly originates in the disharmony of parts, when the individual ones are unable to maintain themselves in due proportion of quantity or quality ; consequently, the first origin of all diseases is an *oportet*—a thing that must be (ein Oportet, das ist, ein gemusst Ding.) The essence of diseases is various, and the doctor who knows not this is blind. The predisposition to diseases, as well as to moral conduct, is often hereditary (aller anfang ist im Vatter). The old doctrine of the four elements and the four cardinal humours is all fudge, and the doctors hid their lies in the humours. The humours are, in truth, born of the disease, not the disease of them. The physician who finds all diseases in the humours prescribes nothing but evacuants. The snow makes not the winter, but the winter the snow, and the winter may last, though the snow be gone. Plants have diseases, but we look not in them for black and yellow bile, neither for melancholia, nor phlegma, nor cholera. The perspiration is salt, pressed from the blood which contains salt, but can a salt be considered a humour?" (p. 99 et seq.)

In his zealous but perhaps justifiable admiration of Theophrastus, Dr. Marx goes on to say that he was the first who laid the foundation of a geographical nosology and medical topography. The following passage expresses the views of a philosophical observer and deep thinker, and could hardly have been penned by one who had not travelled far and seen much :

"Every nation possesses its medicine within itself. I can well imagine that my recipes are ineffectual with foreigners, and those of foreigners with us. I write for Europe; whether my prescriptions will do for Asia or Africa I know not. As every day has its own evil, every religion its own wrong, so has every nation, province, valley, and climate its own form of disease. If every physician were to describe the physical properties of his own locality, and this be done generally, it would be possible to compose a medical work of lands and water as truly as a map of the globe. (p. 106.)

And Paracelsus lost no opportunity of putting his own doctrines into practice. Thus he tells us that calculus is very frequent in Carinthia; while in the Veltlin it, as well as gout, is unknown. (p. 107.)

"We need not expect," says Dr. Marx, "to find any such words as *general therapeutics* in the writings of Theophrastus; the doctrine was of later origin; but the thing itself is there. It is not to be supposed that so thoughtful a physician had not fixed principles on which to operate, or that he examined and knew so well the natural processes of cure without attempting to imitate them. Hear some of his axioms:

"'Merely to cure is not medicine; but well to cure the present and guard against the future malady, that is true art. .......... Nature does not allow herself to be forced or drawn to another course; you must follow her, not she you. If you apply treatment which does not suit her, you injure her......... That is good doctoring, to drive out a disease in the way nature likes: but it is a bad doctoring to dare to get rid of it after your own fashion. .......... Every one should consider that a physician is only the servant of nature and not her master; consequently medicine has to follow the will of nature.'" (p. 107.)

It is impossible to read these axioms without recollecting the almost identical words of the greatest of all philosophers: *Homo Naturæ minister et interpres*, &c.

"The wonderful and almost divine faculty in the living organism of self-preservation and of remedying disease, was recognized by Theophrastus most strikingly in the healing of wounds. Full of admiration of this independent agency, he exclaims 'Not without reason do I call nature the physician of wounds!' (Ich nit unbillich die Natur ein Arzt in der Wunden heiss.) And in the following axioms relating to these and other surgical affections, he exhibits the same thorough knowledge of the nature of sanatory processes: 'Warily must the chirurgeon take heed not to remove or interfere with nature's balsam, but protect and defend it in its working and virtue. It is the nature of flesh to possess in itself an innate balsam which healeth wounds. Every limb has its own healing in itself: Nature has her own doctor in every limb. Wherefore every chirurgeon should know that it is not he, but Nature, that heals.' What do wounds need? Nothing: for inasmuch as the flesh grows from within outwards, and not from without inwards, so the surgery of wounds is a mere defensive, to prevent Nature from suffering any accident from without, so that she may proceed unchecked in her operations.'" (p. 108.)

We suspect that it is only very recently that the generality of even modern surgeons were so thoroughly imbued with the principles on which wounds should be treated; and we fear that there yet exist not a few whose "nimia diligentia" would do well to be schooled in the doctrine of our Theophrastus's balsam. We cannot refrain from quoting yet a few more of these axioms, all showing the same penetration into Nature's secrets:

"In fractures, also, Nature does the whole work herself; all external help being of little moment. Animals show how little need of art there is in these and other cases. The dog licks his wound well; the broken rib of the ox knits of its own accord."

"Time also," he says in another place, "manifests a corresponding power. As long-continued rains at last cease, so there are certain diseases which last long, and when their evil is spent with time, they also stop at last........... Nature knows when she should cure; but not always the doctor; wherefore his business should be to protect nature.... ....... If the doctors would make a clear conscience they must confess that many would get well sooner without them than with them, if they would only turn over their work to Nature."

"We must go back to the first causes of the disease, and not dwell on mere symptoms. We do not endeavour to extinguish smoke, but fire; smoke may indicate fire, but it is not the fire after all. Disease cannot cease, if its cause be not removed." (pp. 109-10.)

The question, also, whether the symptoms of a malady are to be combated by such remedies as produce similar or opposite effects, occupied Theophrastus's attention. He did not coincide with the opinion that every symptom is to be combated by producing the opposite; still he did not adopt that strain of argument which modern homœopathists have carried *ad absurdum*, "That contraria a contrariis curantur, i. e. that heat drives out cold, is false, and never was the case in medicine: but health and disease—they are opposite."

The merits of Theophrastus, as far as the *materia medica* is concerned, were the first to be more generally acknowledged. Theodore Zuinger says of him in his Opera physiol. medica, (Basil. 1610, p. 56:) "Pharmaceuticam subtilem et ingeniosam et efficacem primus Theophrastus P. Helvetius novæ methodi legibus adstrictam, ex latebris chymicis produxit, et ex acroamatica popularem et exotericam fecit." He urges the use of vigorous remedies, and endeavours to understand thoroughly their manner of acting. He recommends simple recipes, proper combination, and an accurate fixing of the time they are to be taken. The main object is to rouse strength; maladies being rather dynamic than material (corpora), we must oppose immaterial to the immaterial. He is much in favour of soothing and quieting remedies, "wherethrough nature, like a man mad with drink, was brought again to reason." He denounced those powerful caustics so much used in his time, viz. corrosive sublimate, white and yellow arsenic, alum with vinegar, vitriolum rubrum ustum, &c. He was perfectly aware of the noxious effects of the abuse of mercury, which, he said, caused dysentery, salivation, cough, phthisis, vehement pains and weakness of the limbs, putrescency. The strength of remedies, he says, it is very difficult to determine. The medicine has to act against disease like fire, and is not a single spark sufficient to burn a wood? consequently the dose of medicine is not of great importance. Here he adopts in full the homœopathic principle. Against the druggists of his time he was very wroth, especially for their playing at medical men; not being aware, he says, that the man who can boil a fish may still be no fisherman!

The following admirable remark is even beyond the most liberal views of our own times: "To spread popular medicine is not only desirable, but a duty. The real aim of medicine is to help the sick, for whose sake the knowledge of remedies should be universal. This would not injure medical men, because experience and tact could never become public property. How much would I bear and suffer," he adds, "to see every one become his own shepherd!" (p. 119.)

Theophrastus knew the heroic drugs as well as any of his contemporaries; and it is because he knew them so well that he used them with the greatest caution. As he, however, liked efficient remedies, and thereby effected great cures, the use of poisons was imputed to him.

This imputation was most probably levelled at his using preparations not generally known. Although he was partial to efficient remedies, he did not deny that there were remedies which could not exist in the form of prescriptions. "The whole world," he says, "is a druggist's shop (*apotheke*); it is but one who manages the pestle all over the globe." He laid also a great stress on diet and regimen, saying "that health must be attended to as well as disease." To the use of water, as well pure as mineral, he ascribed great virtues, and he states that the latter could be made artificially; an assertion which has been strikingly justified in modern times by the successful exertions of his countryman Dr. Struve.

The disease which then most strongly occupied the attention of medical men was syphilis; and as its various forms could not be divined by the common semiotic indications, viz. inspection of urine or the feeling of the pulse, Theophrastus called the then usual proceedings of that kind mere impostures. He says that a person may judge from the urine whether there are stones either in the bladder or the kidneys, and of what sort they are. He recommends to medical men the study of the physiognomy of diseases. He says that he who, through a wrong diagnosis, assumes an evil to exist when it does not, plants one in the body. In reference to these and other proofs of Paracelsus's knowledge of pathology and therapeutics being so superior to that of his contemporaries, Dr. Marx says:

"Whoever will reflect on the previous and then state of these sciences, and compare it with the views and proceedings of Theophrastus, will be compelled to admit that an unusual power, nay, if we may so speak, a sort of inspiration of knowledge of things unheeded of until then, was operating within his mind. Notwithstanding the undigested data he had before him, and other obstacles which he had to encounter, he will appear as a man who, whether understood or not in his own time, has been the forerunner of subsequent beneficial reforms." (p. 127.)

The diseases which occasion some solid deposit of matter, and which Theophrastus calls *tartaric diseases*, occupied much of his attention:

"It is stated," he says, "that there are but two cavities where stones may grow, the bladder and the kidneys; but there are far more, and in every part of the body solid substance may be formed; and these vary with the nature of the locality, a fact of consequence for the physician to know, as he must vary his means accordingly......When sound, the lungs move freely up and down, and receive and emit air; but when the air-passages are obstructed with tartar, then ensue various diseases, variously named by physicians asthma, cough, &c.; but they all are from tartar, and they terminate in phthisis." (p. 135.)

The surgical labours of Paracelsus met with less opposition from his medical contemporaries than did his Medicine. We would gladly enrich our pages with some more of his striking axioms on this head, but our waning space forbids, and we must conclude our hasty and imperfect analysis of this extraordinary production with the following passage, which closes the Memoir of Dr. Marx:

"From his very first outset in public life to his death, the constant aim of Theophrastus was to disseminate his conscientious opinions in spite of all opposition. His memory should therefore be held in honour, and Germany must no longer allow his name to be reviled and scorned: on the other hand the illusion must be abandoned that in his works are to be found proofs of all sorts of knowledge and discoveries. His writings had a great temporary object, and this has been achieved. Neither their form nor the character of their contents make them fit for the study of posterity. The aims of Theophrastus were, to untie the fetters of tradition; to lead the way to new medical truths; to make German physicians conscious of the value of their language, as well as the richness of their own scientific resources, and to oppose every abuse of medical practice. As in the progress of time all these aims (albeit not always in the sense of his intentions and according to the impulse *he* gave) have been realized, and consequently all his wishes and hopes actually accomplished, the sphere of his activity is closed; and history has done enough, preserving gratefully the recollection of his name and deeds."

---

